# Association between braced curve behavior by pubertal growth peak and bracing effectiveness in female idiopathic scoliosis: a longitudinal cohort study

**DOI:** 10.1186/s12891-018-1987-9

**Published:** 2018-03-27

**Authors:** Sai-hu Mao, Xu Sun, Ben-long Shi, Yong Qiu, Bang-ping Qian, Jack C. Y. Cheng

**Affiliations:** 1 Spine Surgery, the Affiliated Drum Tower Hospital of Nanjing University Medical School, Zhongshan Road 321, Nanjing, 210008 China; 2 Joint Scoliosis Research Center of the Chinese University of Hong Kong & Nanjing University, Nanjing, China

**Keywords:** Idiopathic scoliosis, Peak height velocity, Angle velocity, Curve progression, Bracing outcome

## Abstract

**Background:**

Pre-pubertal idiopathic scoliosis (IS) is associated with high risk of bracing ineffectiveness. Integrated multidimensional maturity assessments are useful but complex to predict the high-risk occurrence of curve progression. This study is designed to provide a simple screening method for brace effectiveness by determining whether or not the braced curve behavior at growth spurt, being defined as variations in Cobb angle velocity (AV) at peak height velocity (PHV), can be a new factor predictive of brace outcome prescribed before PHV.

**Methods:**

This is a retrospective study of a series of 35 IS girls with simplified skeletal maturity score no more than 3 at initiation of bracing treatment and followed up through the growth spurt until brace weaning or surgery. Serial Cobb angle and maturity indicators involving height velocity, Risser sign, triradiate cartilage, simplified skeletal maturity score and distal radius and ulna classification were assessed and patients were stratified into either a positive or negative category based on a positive or negative value of AV at PHV. Comparisons were made between the positive and negative AV groups, as well as the failed and successful bracing groups, using independent sample T test and crosstab analysis. Logistic regression analysis was used to identify the predictive factors of failed brace treatment.

**Results:**

Brace treatment prescribed before PHV was found to have an overall failure rate of 57.1% and a surgical rate of 45.7%. Negative AV at PHV accounting for 54.3% of the recruited patients were associated with lower brace failure rate (36.8% vs. 81.2%, *p* = 0.016) and surgical rate (21.1% vs. 75.0%, *p* = 0.002). Patients in the failed bracing group showed higher ratio of thoracic curve (80.0% vs. 26.7%,*p* = 0.002) and higher AV at growth peak (2.3 ± 9.1 vs. -6.5 ± 11.4°/yrs., *p* = 0.016). The logistic regression analysis revealed that positive AV at PHV (OR = 9.268, 95% CI = 1.279–67.137, *p* = 0.028) and thoracic curve type (OR = 13.391, 95% CI = 2.006–89.412, *p* = 0.007) were strong predictive factors of ineffective brace treatment initiated before PHV.

**Conclusions:**

Sustained curve correction following bracing despite early onset and rapid pubertal growth was strongly predictive of effective brace control of scoliosis.

## Background

Idiopathic scoliosis (IS) diagnosed shortly before pubertal growth peak (PHV) is known to have higher probability of rapid curve progression [1–4]. Early asymmetric brace treatment with good compliance is critical at this phase for the indicated patients [5]. However, the reported failure rate for moderate curves braced during the onset and acceleration phase of PHV could be as high as 48% to 83% [6–8], comparing with the 26% reported by Nachemson et al. for patients presented later [9]. Thus the determination of timing relative to peak growth is generally considered to be highly prognostic of the risk of “curve-progression” in IS [4, 10–12].

As a rule-of-thumb, the effect of continuous growth stimulation on curved spine is double-edged, being harmful in untreated patients, and can be inverted to counteract mild to moderate scoliosis progression with the aid of rigid orthosis [13]. Kotwicki et al. reported that vertebral growth is one of the active mechanisms for curve correction in immature patients treated with rigid brace [13]. As in infantile scoliosis, the children’s own growth could be harnessed as a corrective force for straightening the curved spine with the aid of serial plaster jackets [14]. We thus hypothesized that the growth-powering corrective force to remodel the vertebrae and augment the curve correction, if it worked, should be maximum at peak height velocity (PHV). If the corresponding curve behavior, being represented as variation of angle velocity (AV) of braced curve at PHV, showed sustained curve progression, the brace treatment was unlikely to succeed. Thus a close monitoring of braced curve behavior at PHV might be of higher prognostic value in predicting a failed brace treatment in addition to the existing simple maturity assessments, and accordingly be helpful to stratify pre-pubertal IS patients by risk of bracing ineffectiveness.

This longitudinal study is designed to provide a simple screening method for brace effectiveness by determining whether or not the braced curve behavior at growth spurt, being defined as variations in angle velocity (AV) at peak height velocity (PHV), can be a new factor predictive of brace outcome prescribed before PHV.

## Methods

This retrospective longitudinal study was nested in a prospective database of scoliosis patients receiving standardized brace treatment in our hospital since 2007. Ethics approval was obtained from our institutional review board, and patients’ informed consents were obtained prior to commencement.

Study subjects were recruited consecutively with the following eligibility criteria including girls with clinically and radiologically confirmed IS treated with rigid brace and presented with open or semi-open triradiate cartilage, Risser stage 0, pre-menarchal, with the simplified skeletal maturity score (SSMS) no more than stage 3 [4, 10]; Cobb angle at initial diagnosis within 20°-40°; and agreement to receive standardized Milwaukee (for patients with major thoracic or double thoracic curves) or Boston (for patients with double major, thoracolumbar or lumbar curves) brace treatment until brace weaning or surgery [15]. The lower limits of major curve magnitude allowing initiation of brace wear was extended down to 20° due to the great growth potential for this particular patient cohort [16]. For all the recruited patients, full time rigid bracing was prescribed, for 22 h a day. Any patient with poor compliance of brace wear (actual bracing time reported by patients or parents of less than 90% of the prescribed hours) would be excluded from this study [17, 18]. Patients were followed up at 3–6 months interval with serial standing antero-posterior (AP) radiographs of the whole spine without orthosis and the left hand to monitor the change in curve magnitude, brace fitting and skeletal maturity status until the completion of treatment programme beyond skeletal maturity. Corrective surgery was recommended when the curve magnitude of major curve progressed beyond 40–45°, depending on the patients’ maturity status, cosmetic concerns and self-willingness [10, 15]. Weaning of braces would be started when the patients reached Risser sign 4, SSMS stage 7 and 2 years post menarche [10, 15]. The outcome of brace treatment were graded as failure for patients who have ≥6° progression at maturity, or if the patient underwent corrective surgery or reach surgical threshold [15].

### Anthropometric and radiographic measurements

Data acquisitions of relevant clinical and radiographic information were incorporated in the sequential clinic visits. For each follow-up, the chronological age, Cobb angle of major curve, apical Nash-Moe vertebral rotation, standing height, curve pattern, timing of menarche, Risser sign, the status of the triradiate cartilage, the SSMS [10] and the distal radius and ulna (DRU) [11] classification scheme were recorded. The Cobb angle was defined as the angle between the line parallel to the superior endplate of upper end vertebra and the line parallel to the inferior end plate of the lower end vertebra [19], and was obtained after the patient had been out of the brace for minimum 4–5 h. Standing height was measured twice in centimeters by an orthopaedic resident using a wall-mounted ruler with a perpendicular slide, and the mean of both measurements was adopted for analysis. Height velocity (HV) calculations were then performed at a minimal interval of six-months [20], defined as the growth in centimeters per year obtained by dividing the height increase by the time interval between two consecutive medical visits: HV = (Height_n_-Height_n-1_) /(Time interval_n − (n − 1)_) [21]. With these consecutive data, a growth velocity curve was determined for each patient. The age at which the maximum velocity in growth during adolescence was seen was designated as the peak height velocity (PHV).

Determination of Risser sign was based on the modified grading system of United States Risser sign [22]. As for the triradiate cartilage, stage 1 was very widely open, stage 2 was the first sign of osseous invasion of the triradiate cartilage itself, and stage 3 was total ossification [23]. Serial assessments of the SSMS system, as described by Sanders et al. [10], were performed according to the capping and fusion of digital epiphyses, metaphyses and distal radial physis. Standards for how to assess and decide the simplified skeletal maturity system were as follows: Juvenile slow (stage 1): Digital epiphyses are not covered; Preadolescent slow (stage 2): All digital epiphyses are covered; Adolescent rapid-early (stage 3): The preponderance of digits are capped. The second through fifth metacarpal epiphyses are wider than their metaphyses; Adolescent rapid-late (stage 4): Any of distal phalangeal physes are clearly beginning to close; Adolescent steady-early (stage 5): All distal phalangeal physes are closed; Adolescent steady-late (stage 6): Middle or proximal phalangeal physes are closing; Early mature (stage 7): Only distal radial physis is open. Metacarpal physeal scars may be present.; Juvenile slow (stage 1): Digital epiphyses are not covered. The DRU classification scheme was based on the evolution of distal radius and ulna radiological morphology, and mainly covered radius stages R5 to R11 and ulna stages U2 to U9 during puberty [11]. Menstrual status data were collected by inquiring of female participants or their guardians about the age when their periods or menstrual cycles started.

### Assessment of angle velocity variation at peak of growth

Angle velocity was defined as angle change divided by a minimum time interval of six-month between two consecutive or interrupted medical visits, expressed in angle degrees per year: AV = (Angle_n_-Angle_n-1_) /(Time interval_n − (n − 1)_) [24]. For each recruited patient, the angle velocity at growth peak was identified. Patients were then stratified into either a positive or negative category based on the value of AV at PHV.

### Statistical analysis

Statistical analysis was performed using the SPSS software packages 17.0 (SPSS, Inc., USA). Patients’ demographics were analyzed with the descriptive statistics. Data were presented as mean ± standard deviation (SD). Comparisons were made using independent sample T test and crosstab analysis. Logistic regression analysis was used to identify the predictive factors of failed brace treatment. Treatment outcome was coded as 0 for successful bracing and 1 for failed bracing. Magnitude of major curve was coded as 0 for < 30° and 1 for ≥30°. Curve pattern was coded as 0 for thoracolumbar or lumbar curves and 1 for major thoracic curves. AV at PHV was coded as 0 for a negative value and 1 for a positive value. Statistically significant difference was defined as *P* < 0.05.

## Results

Thirty five IS girls fulfilled our inclusion criteria and completed the study. Milwaukee brace was applied to 8 patients with major thoracic curves and 3 patients with double thoracic curves. For 9 patients with double major curves and 15 patients with thoracolumbar/lumbar curves, Boston brace was prescribed. The mean age at the start of brace treatment was 10.5 ± 1.3 yrs. (range, 7.9–13.2 yrs), and the mean follow-up was 4.8 ± 1.7 yrs. (range, 1.3–8.7 yrs). The initial Cobb angle averaged 26.5 ± 5.0° (range, 20°-39°) with 14.3% beyond 30°, and increased to 34.0 ± 12.6° (range, 9°-60°) at final follow up. The apical Nash-Moe vertebral rotation increased from 1.0 ± 0.5 to 1.9 ± 0.8. The mean ages of menarche were 12.1 ± 1.2 yrs.

### Outcome of bracing

The patients with brace treatment prescribed shortly before PHV was found to have a failure rate of 57.1% and a surgical rate of 45.7%. The mean curve magnitude of the failure group increased from 26.4 ± 5.4° to 42.5 ± 8.4°. While for patients successfully treated by bracing, the mean curve magnitude remained stable or decreased measured as 26.7 ± 4.7° at the beginning and 22.8 ± 7.3° at the final visit. The percentage of major thoracic curves was significantly higher in the failure group as compared with the success group (80% vs. 26.7%, *p* = 0.002). In terms of initial curve magnitude, no significant difference was found between the failure and success group (26.4 ± 5.4° vs. 26.7 ± 4.7°, *p* > 0.05) (Table 1).

**Table 1:** Comparison of the baseline demographic and clinical characteristics between the failure and success brace groups

Variables	All patients	Failure	Success	*p* value
Age at diagnosis (yrs)	10.5 ± 1.3	10.3 ± 1.2	10.9 ± 1.3	0.159
Age at final follow-up (yrs)	15.4 ± 2.0	15.3 ± 2.2	15.5 ± 1.6	0.705
Initial curve magnitude (°)	26.5 ± 5.0	26.4 ± 5.4	26.7 ± 4.7	0.857
Final curve magnitude (°)	34.0 ± 12.6	42.5 ± 8.4	22.8 ± 7.3	0.000*^,a^
Final Risser score	3.8 ± 0.9	3.6 ± 1.1	4.0 ± 0.0	0.134
PHV (cm/y)	9.1 ± 1.6	9.0 ± 1.7	9.3 ± 1.4	0.582
Timing of PHV (yrs)	11.6 ± 0.9	11.5 ± 1.0	11.7 ± 0.9	0.684
AV at PHV (°/y)	−1.5 ± 10.9	2.3 ± 9.1	−6.5 ± 11.4	0.016*^,a^
Initial height (cm)	141.9 ± 8.4	140.6 ± 8.6	143.7 ± 8.0	0.237
Final height (cm)	160.4 ± 5.6	159.2 ± 5.8	161.8 ± 5.1	0.176
Percentage of major thoracic curve (%)	57.1	80	26.7	0.002*^,b^

*AV* Angle velocity, *PHV* Peak height velocity

Analyses were performed through

^a^independent sample T test

^b^crosstab analysis

*p < 0.05

The standing height increased from 141.9 ± 8.4 cm to 160.4 ± 5.6 cm throughout the follow-up period, and serial longitudinal measurements identified an average PHV of 9.1 ± 1.6 cm/y, while timing of PHV was recorded as 11.6 ± 0.9 yrs. (Table 1). The timing and magnitude of PHV in the failure group showed no significant difference compared with those in the success group (PHV: 9.0 ± 1.7 cm/y vs. 9.3 ± 1.4 cm/y; timing of PHV: 11.5 ± 1.0 yrs. vs. 11.7 ± 0.9 yrs., *p* > 0.05). The SSMS averaged 3.4 ± 0.6 at PHV and as for radius and ulna epiphysis, the corresponding DRU classification were 8.0 ± 0.7 and 5.9 ± 0.5, respectively. The AV at PHV was significantly larger in the failure group when compared with the success group (AV: 2.3 ± 9.1 °/y vs. -6.5 ± 11.4 °/y, *p* = 0.016).

### Comparison between the subgroups with positive and negative variation of AV at PHV

Negative AV at PHV accounted for a share of 54.3% in the recruited patients, which were associated with lower failure rate (36.8% vs. 81.2%, p = 0.016) and surgical rate (21.1% vs. 75.0%, *p* = 0.002) (Table 2). And accordingly, the final curve magnitude was significantly larger in the positive AV group (39.6 ± 14.2° vs. 29.4 ± 9.1°, *p* = 0.015). There was no difference in timing and magnitude of PHV, as well as curve pattern and initial curve magnitude, between these two groups (*p* > 0.05). Smaller age, smaller initial height and lower degree of initial maturity assessment (SSMS and DRU stages) were detected in the positive AV group (*p* < 0.05).

**Table 2:** Comparison of the baseline demographic and clinical characteristics between the positive and negative AV groups

Variables	Negative AV	Positive AV	*P* value
Failure rate (%)	36.8	81.2	0.016*^,b^
Surgical rate (%)	21.1	75.0	0.002*^,b^
AV at PHV (°/y)	−9.6 ± 7.6	8.2 ± 4.0	0.000*^,a^
Initial curve magnitude (°)	26.3 ± 4.6	26.7 ± 5.7	0.832
Final curve magnitude (°)	29.4 ± 9.1	39.6 ± 14.2	0.015*^,a^
PHV (cm/y)	8.8 ± 1.5	9.4 ± 1.8	0.327
Timing of PHV (yrs)	11.7 ± 1.1	11.5 ± 0.8	0.732
Age at diagnosis (yrs)	11.0 ± 1.3	10.0 ± 1.0	0.014*^,a^
Initial SSMS	2.7 ± 0.6	2.2 ± 0.5	0.006*^,a^
Initial DRU (R)	7.0 ± 0.8	6.4 ± 1.0	0.053
Initial DRU (U)	5.1 ± 0.8	4.3 ± 0.9	0.016*^,a^
SSMS at PHV	3.4 ± 0.5	3.5 ± 0.6	0.495
DRU (R) at PHV	8.0 ± 0.7	8.0 ± 0.7	0.999
DRU (U) at PHV	5.9 ± 0.5	5.8 ± 0.4	0.244
Percentage of major thoracic curve (%)	47.3	68.8	0.306
Initial height (cm)	145.2 ± 7.2	138.3 ± 8.2	0.013*^,a^
Final height (cm)	161.3 ± 5.4	159.2 ± 5.6	0.257

*AV* Angle velocity, *PHV* Peak height velocity, *SSMS* Simplified skeletal maturity score, *DRU* Distal radius and ulna classification

Analyses were performed through

^a^independent sample T test

^b^crosstab analysis

*p < 0.05

### Results of logistic regression analysis

**Fig. 1:**
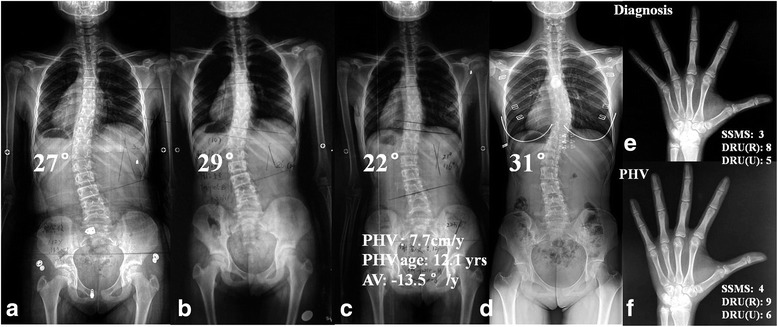
an adolescent girl, with major thoracolumbar IS at diagnosis (Cobb angle: 26°, curve apex: T12, SSMS 3, DRU(R) 8 and DRU (U) 5) (**a**, **e**). Full time Boston brace was prescribed, and the major Cobb angle kept being slowly progressive (**b**). The timing and magnitude of PHV was 7.7 cm/y and 12.1 years old, respectively (**c**). And the corresponding staging of SSMS, DRU (R) and DRU (U) were 4, 9 and 6, respectively (**f**). The AV decreased to − 13.5°/y by PHV, resulting in temporary curve resolution. By skeletal maturity aged 16.6 yrs., the major curve grew to 31° (**d**) and the brace treatment was considered successful

**Fig. 2:**
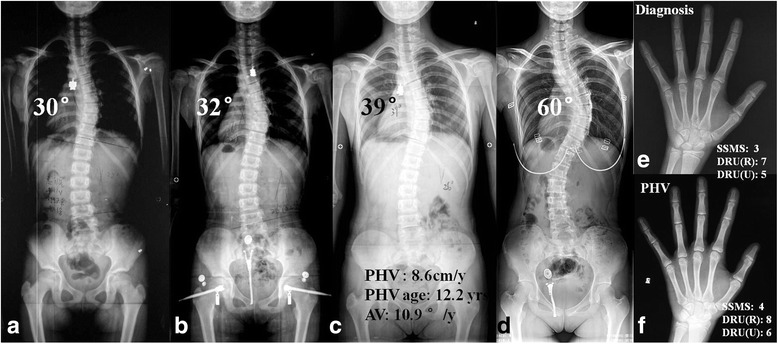
an adolescent girl, with major thoracic IS at diagnosis (Cobb angle: 30°, curve apex: T9, SSMS 3, DRU(R) 7 and DRU (U) 5) (**a**, **e**). Full time Milwaukee brace was prescribed, yet the major Cobb angle kept being slowly progressive (**b**). The timing and magnitude of PHV was 8.6 cm/y and 12.2 years old, respectively (**c**). And the corresponding staging of SSMS, DRU (R) and DRU (U) were 4, 8 and 6, respectively (**f**). The AV accelerated rapidly and by PHV it reached 10.9°/y, resulting in continuous curve deterioration. By age 16.2 yrs., the thoracic curve grew to 60° (**d**) and corrective surgery was recommended

The logistic regression analysis revealed that positive AV at PHV (OR = 9.268, 95% CI = 1.279–67.137, *p* = 0.028) and thoracic curve type (OR = 13.391, 95% CI = 2.006–89.412, *p* = 0.007) were strong predictive factors of failed brace treatment initiated before PHV (Table 3). In contrast, the curve magnitude at diagnosis could not be retained in the model (*p* = 0.382). Examples of the representative association between AV variations at PHV and bracing outcome were shown in Figs. 1 and 2, respectively.

**Table 3:** Results of logistic regression analysis

	Regression coefficient	*p*	Odds ratio	95% CI
AV at PHV	2.227	0.028	9.268	1.279–67.137
Curve pattern	2.595	0.007	13.391	2.006–89.412
Cobb angle	0.885	0.382	2.423	0.333–17.638

CI=Confidence Interval

Treatment outcome was coded as 0 for successful bracing and 1 for failed bracing. Magnitude of major curve was coded as 0 for < 30° and 1 for ≥30°. Curve pattern was coded as 0 for thoracolumbar or lumbar curves and 1 for major thoracic curves. AV by growth peak was coded as 0 for a negative value and 1 for a positive value

## Discussion

The use of rigid brace to slow or halt curve progression is a time-honored therapeutic strategy worldwide [25]. Early or immediate bracing for patients presented before PHV in a growing spine with curve above 20–25 deg. has been the consensus [13, 15, 26, 27]. In the absence of effective bracing, the rapid growth in the at risk group could lead to accelerated curve progression [28]. However, Kotwicki et al. [13] also pointed out that the vigorous and asymmetrical vertebral growth could be harnessed as one of the active mechanisms promoting the positive outcome of bracing. The concave unloading and remodeling of the vertebrae assisted by orthosis might have accounted for this corrective capability [13]. So, for patients with comparable high level of immaturity, the current dilemma lied in how to identify those that would benefit effectively from these active bracing mechanisms. We believed that such growth-related modifying effect would be most profound by peak of growth and that the responsive curve behavior represented by angle velocity at PHV might be an important predictor of the brace outcome, a factor which unfortunately hadn’t been adequately studied.

In this study cohort, all patients started bracing before growth peak. The follow-up results showed a failure rate of 57.1% and a surgical rate of 45.7%. The overall poorer results when compared to those in more matured patients were consistent with a number of other reports [9, 18]. Khoshbin et al. [6] reported that in patients with juvenile IS who started bracing at an age averaged 9.3 yrs., the incidence of curve progression reached 72%, and the surgical rate was as high as 50%. An even longer growth period for juvenile patients along with greater initial curve magnitude in his study might account for such discrepancy. Little et al. [7] also revealed a progressive rate of 73.3% and a surgical rate of 42.5% for patients braced through pubertal growth spurt.

Despite the poor bracing outcome in this series, this study was unique in showing that the AV was not always positively proportional to linear growth velocity in braced curves. And the proportion of negative AV at growth peak, being representative of curve correction, could reach as high as 54.3%. The logistic regression analysis further revealed that patients with negative AV at PHV were more responsive to rigid orthosis, with an odds ratio of 9.27. These data gave rise to the concept that variations of AV at PHV could be a new factor representative of the growth-powering corrective capability of each IS patients, regardless of the curve pattern, magnitude and stiffness, thus being strongly predictive of the pubertal bracing outcome.

This new predictive factor could have important potential clinical application and provided a better understanding of variations of braced curve behavior during rapid growth peak. The curve reduction, introduced by bracing, might enhance the remodeling activity of the growing spine [14] through the passive “cherry stone” effect [13], elongate the spine and unload the concave half of the apical vertebrae. The vertebral growth could then act as a corrective force and break the vicious cycle of growth asymmetry in the framework of the Heuter-Volkman law [29, 30], thus allowing structural remodeling of the wedged vertebrae [13, 31]. Moreover, the continuous application of an external orthosis could guide the vertebral growth into more physiological alignment three dimensionally [14] and help to stabilize or straighten the curved spine.

Accordingly, we believed that patients with a negative AV at PHV should be advised to continue with full time rigid brace to achieve the best outcome. Braced patient with positive angle velocity at the PHV should be well informed of the high risk of surgical intervention in addition to the strict bracing program. This prognostic prediction could be further enhanced by coupling with the multiple simplified skeletal maturity assessment methods which could help to predict the onset of peak height velocity [10, 11]. The SSMS stage 3, which was characterized by preponderance of capped digits and the second to fifth metacarpal epiphyses, was reported by Sanders to strongly signify the onset of PHV [10]. Moreover, in Luk’s new DRU classification, characterization involving the medial capping of the distal radius, the appearance of the ulna styloid and the head of the ulna being distinctly defined and denser than the styloid was highly indicative of growth peak [11]. These additional information could further help in clinical-decision making and management.

For patients with brace prescribed in post-PHV pubertal stages, typically at Risser 1 or 2, without the additional information of AV at PHV, other prognostic factors would need to be taken in consideration in guiding the clinical management. Poor initial correction in brace ranging from 10%–40% was found to be associated with higher failure rate [32–35]. The initial reduction in angular velocity was reported to be better than the initial Cobb angle correction rate in predicting the outcome of bracing. A reduction of lower than 10°/year was found to be highly predictive of bracing failure [36]. This study also showed that thoracic curve tended to be more resistant to brace treatment with poorer outcome, a finding consistent with many other reports [1, 37, 38]. The role of initial curve magnitude as a risk factor was not clear in the logistic regression analysis of the current study probably related to the relatively smaller series in contrast to some previous studies that generally showed a poorer outcome with threshold level of 30° [1, 10].

A methodological limitation of this study might be the relatively small sample size. However, the meticulous prospective data collection and no loss of follow up allowed sufficient comparison of the two groups. Another limitation lied in that the bracing compliance was not monitored with either a pressure or temperature sensor, which might be a point of focus in further studies since Aulisa et al. reported that the incidence of curve progression are lower in patients with high brace compliance [39].

## Conclusion

The current study, to our best knowledge, was the first study focusing on investigating the predictive value of curve behavior at PHV on bracing outcome. A positive AV at PHV indicated sustained curve progression being irreversible despite strict bracing and maximal growth-powering corrective force at growth peak, and thus was less likely to respond effectively to brace control of scoliosis, especially for patients with major thoracic scoliosis. Consequently, surgery would likely to be a plausible treatment. Otherwise, sustained curve correction following bracing despite early onset and rapid pubertal growth was strongly predictive of effective brace control of scoliosis. In this situation, continuous full time rigid brace were necessary to maximize bracing effectiveness over the time. This preliminary screening information was essentially instructional and would assist in counseling pre-pubertal scoliosis patients in regards to their concerns with prognostication and management of scoliosis. The aim of brace treatment could thus be roughly stratified into either curve control or delay of the time point of surgical intervention. With further larger series validations across different centers, it was hoped that the AV at PHV could be used to predict and stratify patients for risk of curve progression and bracing outcome. At this stage, it is clear that accurate prediction of brace outcome should still be approached by employing as many of the available predictive risk factors as possible.

## Abbreviations

AV: angle velocity
DRU: distal radius and ulna; n, number
HV: Height velocity
IS: idiopathic scoliosis
PHV: peak height velocity
SSMS: simplified skeletal maturity score
